# Fibrin, Albumen, Casein, as Elements of Nutrition

**Published:** 1842-04

**Authors:** 


*Fibrin, Albumen, Casein, as Elements of Nutrition.* By Prof. Liebig.—These substances have this character in common, that they all comport themselves alike in regard to concentrated hydrochloric acid. They all dissolve in this acid with the aid of heat, and, kept for a time in a higher temperature, first assume a beautiful lilac, and then a rich violet blue color. At this stage of the decomposition, each of the three substances reacts in the same way with carbonate of ammonia, and other reagents. Repeated analysis in the laboratory of Liebig, has in fact shown, that these three substances have the same elementary composition. The gases obtained by burning these substances with oxide of copper, consist in eight parts by volume of seven volumes of carbonic acid, and one volume azotic gas. The dried flesh of the ox, the deer, the cod and pike, and blood do not differ in composition. Extending our inquiries to the azotized articles of food consumed by phytivorous animals and man, vegetable fibrine (gluten), vegetable albumen, and vegetable casein (a substance which is contained abundantly in all oily seeds), we find that these substances have precisely the same composition as the equivalent animal proximate principles; they consist of eight volumes, seven volumes being carbonic acid, and one volume nitrogen. There is, therefore, absolutely no difference between the azotized elements of nutrition made use of by the phytivorous and the carnivorous tribes of animals.

But a large proportion of substances used as food by phytivorous animals—sugar, gum, starch—contain no azote, and cannot minister to the formation of muscles, nerve, &c.—what purpose do they serve in the economy? They are in the first place, by the loss of an atom or so of oxygen, converted into fat, and the question now comes to be, what is the object of the fat which we see so generally accumulated among animals? At every period of life, man and animals are exposed to ceaseless causes of decay, through the influence of the air of the atmosphere. With every breath, man parts with a portion of his body; in every moment of his life he produces a quantity of carbonic acid, the carbon of which must be replaced by that which is contained in the articles consumed as food.

Now, if we observe an animal or a man in circumstances in which he takes no food, in the state of disease when the body is not renewed, when it has not that restored to it from without, which it loses in the course of its own vital processes, we see that the fat immediately begins to disappear, that the body wastes. The fat, in fact, escapes through the skin and from the lungs, in the shape of carbonic acid and water; we find no trace of it either in the feces or the urine; it serves as the means of withstanding the influence of the atmosphere; it serves for the defence of the various organs. With the disappearance of the fat, however, the influence of the atmosphere does not cease; the other soluble parts of the body replace the fat, and yield their carbon in the struggle. At length all resistance ends, death ensues, and, putrefaction commencing, all the parts of the body enter into combination with the oxygen of the atmosphere. In the greater number of diseases that are called chronic, death follows through the influence of the atmosphere—through a want of resistance against the oxygen of the air. It is the carbon of the vital organs, of the nerves and cerebral mass in especial, that is expended in this direction. In the normal or healthy state, the carbon that is expended in the production of carbonic acid, must needs be derived from other sources.—*London and Edinburg Monthly Journal of Medical Science*, Nov. 1841, from *Ann. der Chemie und Pharmacie*, Aug. 1841.

